# 35 Real-world Experience of Anacaulase-bcdb Debridement in Burns

**DOI:** 10.1093/jbcr/iraf019.035

**Published:** 2025-04-01

**Authors:** Cameron Gibson, Emily Dorgan, Scott Mueller, Arek Wiktor

**Affiliations:** University of Colorado; University of Colorado; University of California Health; University of Colorado

## Abstract

**Introduction:**

Determining the need for surgery can be difficult during the first few days, often requiring a period of observation as the burn “declares” its true depth. Enzymatic debridement (ED) using anacaulase-bcdb was only recently approved by the United States Food and Drug Administration in 2023. The purpose of this study is to compare real-world use outcomes from our first cohort of patients treated with this novel ED agent to historical control patients.

**Methods:**

This retrospective cohort study compared patients treated with anacaulase to 1:1 match controls at an ABA verified burn center from November 2023 to August 2024. Control patients were identified from a cohort admitted to the burn unit in 2023 with burns of indeterminate depth prior to availability of ED. Demographic, clinical, and photographic data were collected to ensure appropriate matching. Outcomes of interest included: hospital length of stay (LOS), need for and number of surgeries, time to first surgery, autograft size (cm2), total opioid and benzodiazepines received for wound care over the first 5 days, pain and sedation scores during wound care, time to 95% wound closure, and readmission data. Descriptive statistics were used to assess institutional anacaulase treatment practices while non-parametric tests were used for all comparisons.

**Results:**

13 ED patients were identified with a non-ED match. Median (interquartile range) cohort age, total body surface area (TBSA), and mechanism of injury were 46 years (33.5, 59.5), 4.5% (2, 10.2), and flame (46.2%) followed by flash (42.3%). No baseline differences were identified between groups. ED was used on or before day 2 with regional anesthesia in all but one case and successfully removed eschar in 92.3%. Median fentanyl and IV midazolam equivalents, maximum pain score, and minimal RASS were 150mcg, 2.5mg, 7, and -1, respectively during ED. Patients treated with anacaulase compared to control had a shorter time to first surgery from admission (4 days [3, 5] vs 6 [5, 7], p=0.017) and higher average maximum wound care pain scores during the first 5 days (7 [6, 8] vs 5 [3, 7], p=0.047). There was no difference in length of stay, area grafted, need for surgery, number of surgeries, total opioids/benzodiazepines received, or RASS scores.

**Conclusions:**

ED of indeterminate depth burns with anacaulase was effective in removing burn eschar and was associated with increased pain during wound care and a shorter time to surgery. Hospital LOS, need for and number of surgeries were not different between groups.

**Applicability of Research to Practice:**

This study demonstrates that ED of indeterminate depth burns with anacaulase can help burn care providers determine a burned patient’s surgical requirements earlier. Providers should have a pain protocol in place to address the increased pain associated with this novel agent.

**Funding for the Study:**

This work was supported by a small investigator-initiated industry grant. Investigator maintains independence over study design analysis and interpretation.

